# Bladder augmentation using a duplicated colonic segment in a patient with a skin-covered variant of classic bladder exstrophy: A case report

**DOI:** 10.1016/j.eucr.2026.103656

**Published:** 2026-10-25

**Authors:** Craig Cronin, Susanna Ezeh, Marjorie Johnson, Catherine Robey, Logan Galansky, Heather Di Carlo

**Affiliations:** a The James Buchanan Brady Urological Institute, Johns Hopkins School of Medicine, Baltimore, MD, USA; b Johns Hopkins University, Baltimore, MD, USA

**Keywords:** Bladder augment, Duplicated colon, Bladder exstrophy

## Abstract

Covered variant is a rare form of bladder exstrophy where a portion or all of the bladder is covered with skin. We present the longitudinal surgical care of a 12-year-old male with concomitant skin-covered exstrophy variant, bifid phallus, and an incidentally discovered duplicated descending colonic system. He underwent elective continence procedure consisting of bladder augmentation utilizing the duplicated bowel segment, bladder neck transection and Mitrofanoff appendicovesicostomy. This case emphasizes the need for multidisciplinary care in the continuing management of complex bladder exstrophy cases.

## Highlights

• Case of bladder exstrophy variant with duplicated colonic segment
• Duplicated colonic segment utilized for augmentation cystoplasty
• Multidisciplinary continence procedure with pediatric colorectal and urology

## 1 Introduction

Bladder exstrophy is a complex congenital urologic condition affecting approximately 1 in 50,000 live births, with a 2:1 male predominance.^1^ Skin-covered exstrophy, represents a rare phenotypic variant of the exstrophy-epispadias complex characterized by partial or complete cutaneous and subcutaneous coverage of the bladder in addition to features like pubic diastasis.^2^ This rare presentation has been primarily described in case reports and is often associated with external genital anomalies, such as bifid phallus and anorectal malformations, which are uncommon features of classic bladder exstrophy.^2^

Initial surgical management of bladder exstrophy typically involves single or staged initial bladder reconstruction. The need for additional continence surgery is often guided by native bladder growth and dry intervals and often includes bladder neck reconstruction or transection with creation of a continent catherizable channel.^3^ Augmentation cystoplasty may also be required in patients with inadequate bladder capacity or poor compliance and is most commonly performed using a segment of ileum.^3^ This case report describes the longitudinal surgical care of a young male with skin-covered exstrophy, concluding with his unique continence procedure that utilized a duplicated colon for bladder augmentation.

## 2 Case presentation

This is a 12-year-old male with a history of skin-covered bladder exstrophy variant, rectal prolapse, and an incidentally discovered duplicated colon. Initial closure of his variant exstrophy occurred in March 2014, and he underwent an elective continence procedure in June of 2026 that included combined urologic and colorectal reconstruction.

The patient was born full-term in 2013. Initial exam was notable for a 4.58 cm pubic diastasis, a small-capacity bladder (10 mL) located beneath the umbilicus and pelvic skin, a patent opening above a bifid phallus, bilaterally descended testes with a right hydrocele, bilateral inguinal hernias (Fig. 1A). He first underwent bilateral inguinal hernia repair and right hydrocelectomy in July 2013. Subsequent evaluation demonstrated a 5.1 cm pubic diastasis and a bladder capacity of 15 mL with bilateral grade 2 vesicoureteral reflux (VUR) (Fig. 1B). In March 2014, he underwent closure of his bladder exstrophy variant with patent urachal repair, umbilicoplasty, epispadias repair with Mustardé urethroplasty, and bilateral anterior and posterior iliac osteotomies with external fixation. Initial postoperative recovery was uncomplicated.

**Fig. 1:**
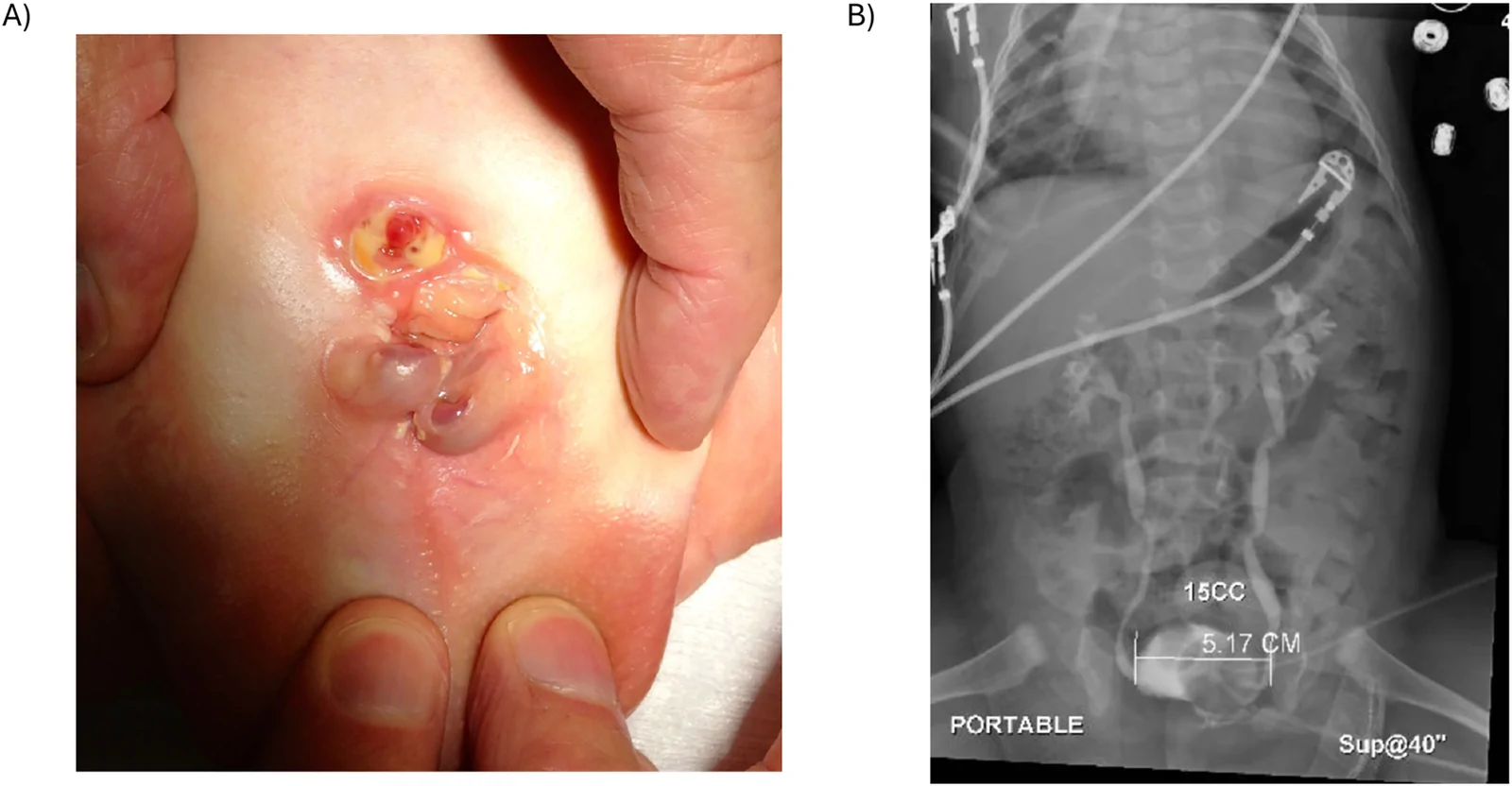
Bladder exstrophy with bilateral vesicoureteral reflux A) Abdominal appearance at birth B) Cystoscopy cystogram demonstrating a small-capacity bladder (approximately 15 mL) with bilateral grade 2 vesicoureteral reflux and 10 Fr catheter in place.

Subsequently, he developed a urethral stricture at the perineal urethral anastomosis. He underwent repeated dilations in 2014 and 2015. In 2016, he underwent perineal urethroplasty with rotational vascularized penile skin flaps to unite the penile halves. Over the following years, he underwent serial cystoscopy and cystograms to monitor bladder growth, VUR, and urethral anatomy. His bladder capacity gradually increased from 39 mL in 2015 to 74 mL by 2019. He continued to have intermittent bilateral or left unilateral grade 2-3 reflux. Given persistently limited bladder capacity, he underwent bladder neck bulking agent injection in 2019. After this procedure, his capacity increased to 118 mL by 2020.

Continued surveillance from 2021 to 2025 demonstrated bladder capacities ranging from 134 to 160 mL, with resolution of VUR by 2025. His clinical course was also notable for long-term trimethoprim-sulfamethoxazole prophylaxis for VUR, recurrent epididymitis, persistent urinary incontinence requiring pads, and chronic constipation with rectal prolapse. Given his rectal prolapse, he was evaluated by pediatric surgery prior to undergoing a continence procedure. Contrast enema identified a duplicated/bifurcated colonic segment arising at the splenic flexure, coursing inferiorly to the descending colon, and terminating near the sigmoid colon (Fig. 2A). Based on these findings, a combined reconstructive approach was planned, including bladder augmentation and creation of a catherizable channel creation with concomitant colorectal reconstruction and rectopexy.

**Fig. 2:**
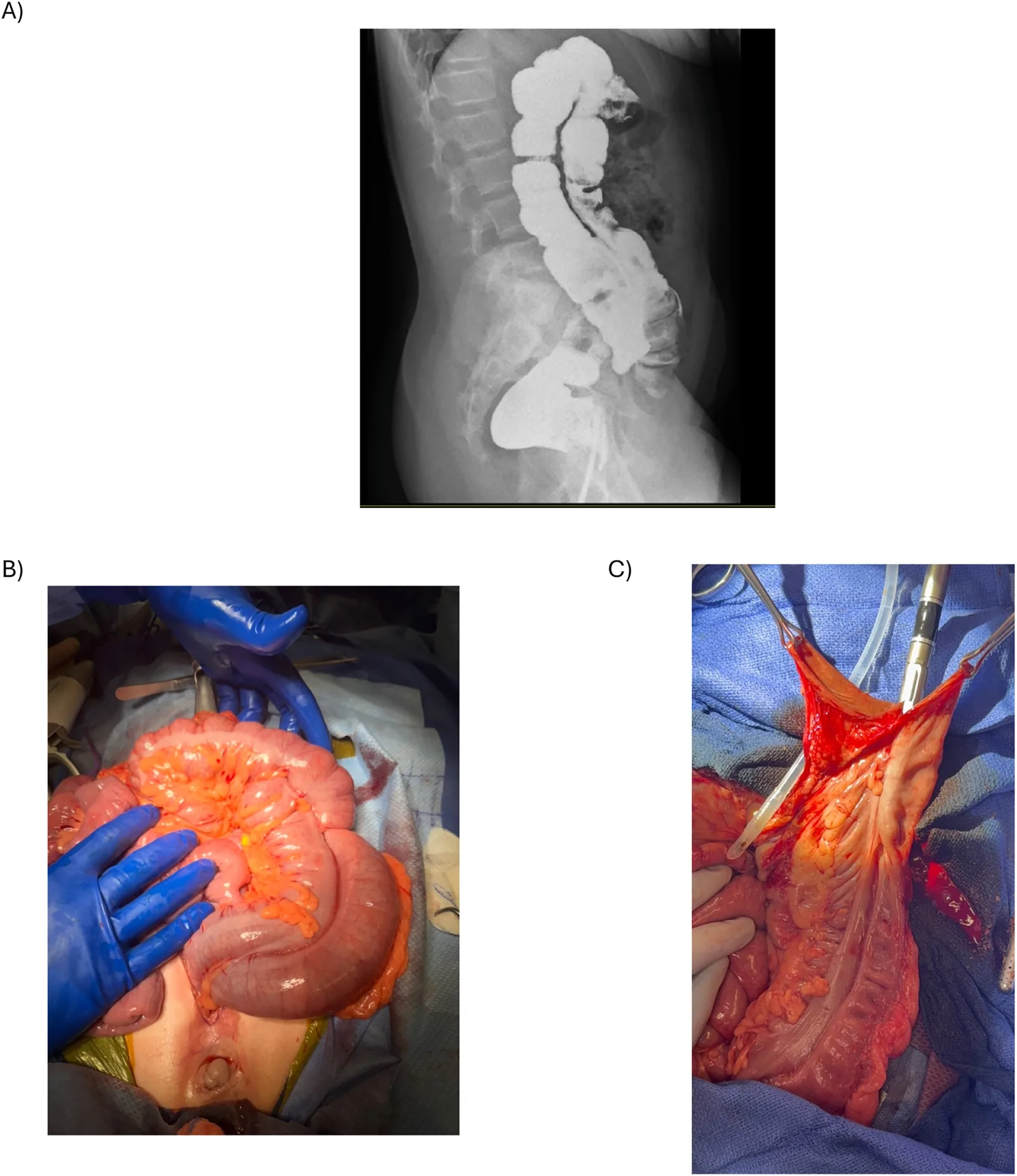
Duplicated colonic segment used for bladder augmentation; A) Contrast enema demonstrating colonic bifurcation at the splenic flexure coursing inferiorly to the descending colon toward the sigmoid colon B) Intraoperative exposure of the duplicated colonic segment C) Longitudinally opened and stapled colonic segment prepared for incorporation into the bladder augmentation.

In June 2026, he underwent his planned continence procedure. The colorectal team identified the duplicated left colon, harvested a 28 cm colonic segment for urinary reconstruction, and performed a coloplasty of the duplicated segment by stapling across the common wall with rectopexy for rectal prolapse (Fig. 2B and C). Intraoperative sigmoidoscopy showed the duplicated left colon originating from the distal transverse colon, which shared a common mesenteric blood supply with the native colon and terminated in a blind-ending pouch. The urologic portion of the procedure included bladder augmentation cystoplasty using the reconstructed duplicated colonic segment, bladder neck transection, Mitrofanoff appendicovesicostomy, suprapubic tube placement, and left vasectomy for treatment of recurrent epididymitis. The appendicovesicosotomy was anastomosed to the native bladder with the duplicated colonic segment prepared for augmentation (Fig. 3). The urethral stump was closed in multiple layers with biologic graft reinforcement. A pedicled omental flap was interposed between the urethral stump and the transected bladder.

**Fig. 3:**
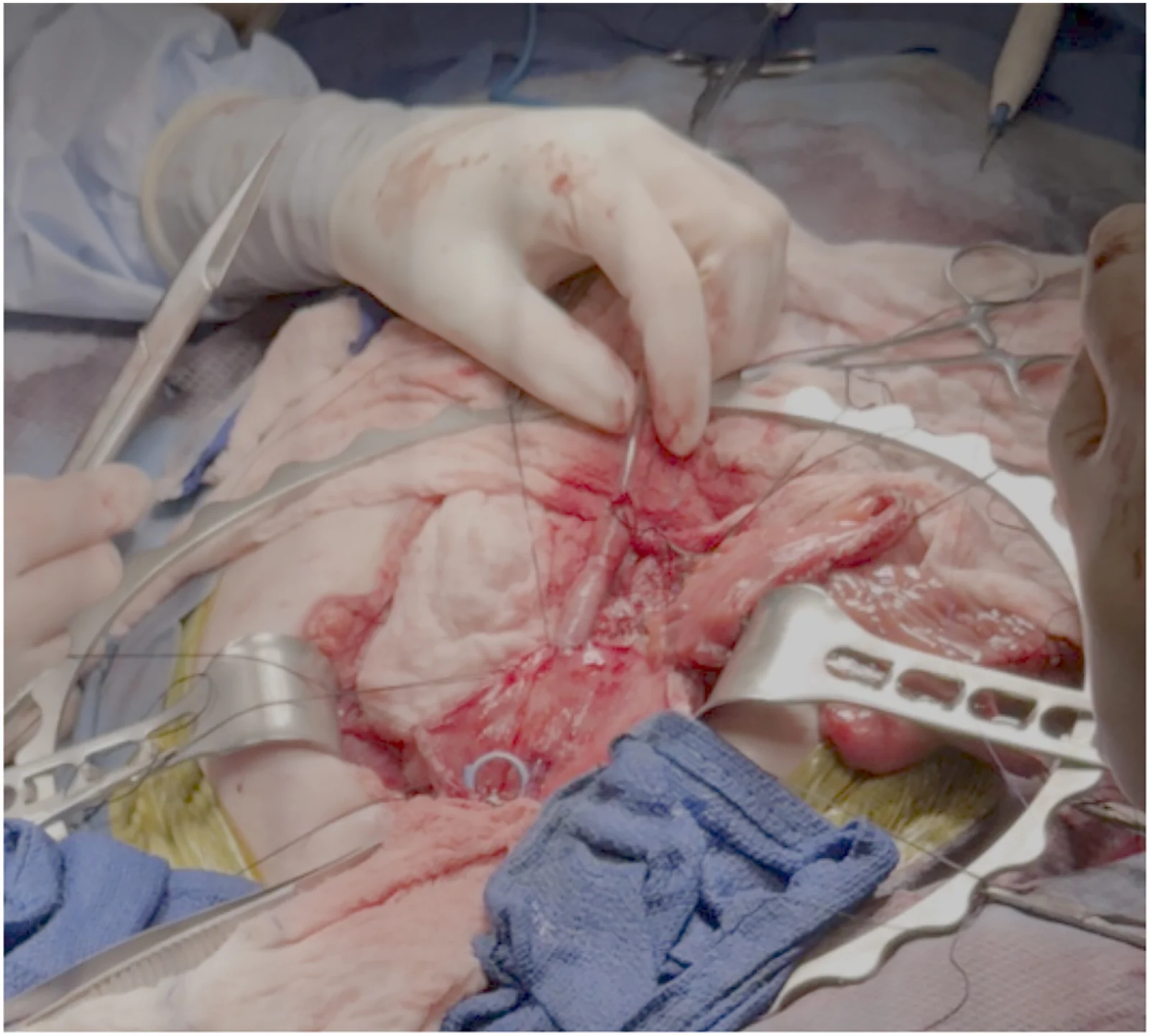
Mitrofanoff appendicovesicostomy and preparation for bladder augmentation. Intraoperative view demonstrating the appendicovesicostomy anastomosed to the native bladder, with the duplicated colonic segment positioned for subsequent bladder augmentation.

Postoperatively, he had a suprapubic tube, stoma catheter, nasogastric tube, Jackson-Pratt drain, and incisional wound vac in place. His post-operative course was complicated by ileus and recurrent emesis. CT scan was completed for evaluation of bowel obstruction and was notable for a large stool burden. He was treated with temporary bowel rest. His diet was then gradually advanced. By discharge on postoperative day 16, he was tolerating a regular diet without nausea or vomiting. Since discharge, he has recovered appropriately, reporting intermittent leakage around the stoma catheter during irrigation and significant mucus production requiring repeated irrigations.

## 3 Discussion

Skin-covered variants represent a rare presentation of the exstrophy-epispadias complex and may be associated with congenital anomalies seen in classic exstrophy as well as anorectal malformations.^2^ Following exstrophy closure, many patients ultimately require continence procedures to achieve dryness. The timing and choice of reconstruction should be individualized based on factors including bladder capacity, the presence and severity of VUR, and, importantly patient readiness and maturity for clean intermittent catheterization. We describe the first reported use of a surgically excised duplicated colonic segment for augmentation cystoplasty.

Skin-covered variants are thought to share the same underlying embryologic mechanism as other forms of the exstrophy-epispadias complex, arising from abnormal caudal mesodermal migration and development of the cloacal membrane.^4^ Incomplete rupture of the cloacal membrane allows the bladder to remain covered by skin, while associated musculoskeletal defects persist. Nearly all reported cases demonstrate pubic diastasis with divergent rectus muscles.^2^ In contrast to classic exstrophy, epispadias is uncommon, and many patients have a functionally normal and continent lower urinary tract.^2^, ^5^ Several reports have also described associated colonic and anogenital malformations, including sequestered bowel, pouch colon, and imperforate anus.^2^, ^5^, ^6^, ^7^.

Although a concomitant duplicated colonic segment has not previously been reported with skin-covered exstrophy, its presence in this case may represent a shared embryologic disturbance or a coincidental anomaly. Intestinal duplications are rare, occurring in approximately 1 in 4500 individuals, and may arise anywhere along the gastrointestinal tract. However, they most commonly occur in the small bowel.^8^, ^9^ Their pathogenesis remains unclear, with proposed mechanisms including prenatal vascular insult, persistence of embryonic diverticula, and failure of intestinal recanalization.^8^, ^10^ Intestinal duplications typically arise along the mesenteric border and often share the muscular wall and blood supply of the adjacent bowel.^11^ In this case, the patient was largely asymptomatic. His rectal prolapse and persistent constipation were what prompted escalation of management and contrast enema which incidentally discovered a bifurcated colonic segment originating near the splenic flexure. Multidisciplinary planning with both pediatric colorectal surgery and urology allowed the use of the duplicated colonic segment for bladder augmentation which avoided ileal resection.

In the absence of a duplicated colonic segment, ileocystoplasty and sigmoid cystoplasty are among the most commonly used techniques for bladder augmentation in patients with bladder exstrophy. Both approaches can effectively increase bladder capacity and compliance, although each carries distinct considerations, including metabolic consequences, mucus production, and the potential for stone formation.^12^, ^13^, ^14^, ^15^ To our knowledge, this is the first reported use of a duplicated colonic segment for augmentation cystoplasty. The patient and family were counseled on the expected risks of use of a colonic segment, including increased mucus production and possible stone formation. Use of the duplicated colonic segment also avoided complications associated with ileal resection, such as bile acid malabsorption and vitamin B12 deficiency.^12^ Although colonic augmentation has historically been associated with an increased risk of urolithiasis, this risk may be less pronounced than previously thought, as the largest reported series demonstrated comparable stone rates across bowel segments used for augmentation.^16^ Given the availability of a large duplicated colonic segment, the opportunity to preserve the remainder of the native intestinal tract, and prior literature supporting the efficacy of sigmoid segments for augmentation, the duplicated segment was selected for cystoplasty. Early postoperative outcomes suggest that this approach is viable, safe, and ultimately effective.

## 4 Conclusions

This case underscores the value of individualized, multidisciplinary planning in the surgical management of complex bladder exstrophy cases. To our knowledge, this is the first reported use of a duplicated colonic segment for augmentation cystoplasty, suggesting that duplicated bowel may serve as a viable reconstructive option.

## CRediT authorship contribution statement

**Craig Cronin:** Conceptualization, Investigation, Writing – original draft, Writing – review & editing. **Susanna Ezeh:** Writing – original draft. **Marjorie Johnson:** Writing – review & editing, Conceptualization, Supervision. **Catherine Robey:** Writing – review & editing. **Logan Galansky:** Writing – review & editing. **Heather Di Carlo:** Project administration, Supervision, Writing – original draft, Writing – review & editing, Visualization.

## Funding

This research did not receive any specific grant from funding agencies in the public, commercial, or not-for-profit sectors.
